# Utilizing cell-free DNA to predict risk of developing brain metastases in patients with metastatic breast cancer

**DOI:** 10.1038/s41523-023-00528-z

**Published:** 2023-04-19

**Authors:** Neelima Vidula, Andrzej Niemierko, Katherine Hesler, Lianne Ryan, Beverly Moy, Steven Isakoff, Leif Ellisen, Dejan Juric, Aditya Bardia

**Affiliations:** Massachusetts General Hospital Cancer Center, Boston, MA 02114 USA

**Keywords:** Breast cancer, Cancer genomics

## Abstract

We compared cell-free DNA (cfDNA) results at MBC diagnosis in patients who developed brain metastases (BM) vs those without (non-BM) to understand genomic predictors of BM. Patients with cfDNA testing at MBC diagnosis (Guardant360®, 73 gene next generation sequencing) were identified. Clinical and genomic features of BM and non-BM were compared (Pearson’s/Wilcoxon rank sum tests). Eighteen of 86 patients (21%) with cfDNA at MBC diagnosis developed BM. Comparing BM vs non-BM, a higher prevalence of *BRCA2* (22% vs 4.4%, *p* = 0.01), *APC* (11% vs 0%, *p* = 0.005), *CDKN2A* (11% vs 1.5%, *p* = 0.05), and *SMAD4* (11% vs 1.5%, *p* = 0.05) was observed. Seven of 18 BM had ≥1 of the following 4 mutations in baseline cfDNA: *APC, BRCA2, CDKN2A* or *SMAD4* vs 5/68 non-BM (*p* = 0.001). Absence of this genomic pattern had a high negative predictive value (85%) and specificity (93%) in excluding BM development. Baseline genomic profile varies in MBC that develops BM.

## Introduction

Metastatic breast cancer (MBC) can vary in disease presentation, subtype, and molecular profiles, and these variable features may profoundly impact patient outcomes^1–3^. Brain metastases in patients with MBC cause significant morbidity and mortality^4^, and may affect a proportion of patients with breast cancer^5,6^. Some predictors for the development of brain metastases such as disease subtype, presence of lung metastases, and extensive visceral involvement are well-documented^7^. Prior studies have demonstrated that genomic profiles may vary in breast cancer, contributing to disease presentation and outcomes^8–13^. However, specific genomic predictors of brain metastases in patients with MBC are not well-understood, given that brain tumor tissue is not routinely obtained for genotyping due to the difficulty of obtaining brain tissue via surgical procedures. In recent years, cell-free DNA (cfDNA) has emerged as a strategy to identify tumor mutations of therapeutic and prognostic significance^14–18^, and offers the advantage of being much less invasive than a tumor tissue biopsy. It is not known whether cfDNA could potentially be used to help identify patients with MBC who may be at higher risk for the development of brain metastases. Understanding potential genomic risk factors for the development of brain metastases could help provide rationale for studying screening and/or therapeutic interventions to reduce the burden of brain metastases in genomically high-risk populations.

The purpose of this study was to explore the potential utility of cfDNA for the identification of patients with MBC with a high risk of developing brain metastases. We compared tumor genotyping results via cfDNA collected at MBC diagnosis in patients who developed brain metastases after MBC diagnosis (BM) with those who did not develop brain metastases (non-BM) to understand potential differences in baseline genomic characteristics.

## Results

### Clinical characteristics of BM vs non-BM

**Fig. 1:**
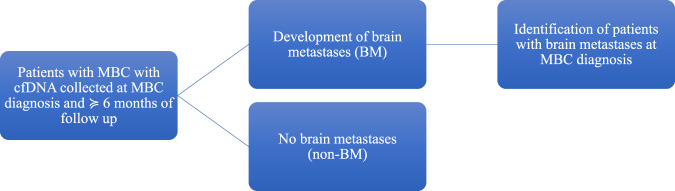
Consort diagram of study cohorts. In this study, patients with MBC with cfDNA collected at MBC diagnosis with ≥6 months of follow up were analyzed for the development of brain metastases (BM). Those patients who had BM at diagnosis of MBC were also identified.

Of 86 patients with MBC who had cfDNA collected at the time of MBC diagnosis, 18 (21%) developed brain metastases during their disease course, of which 5 patients had brain metastases at the time of MBC diagnosis (Fig. 1). Table 1 depicts the baseline characteristics of both cohorts. The median time to development of brain metastases after cfDNA testing was 11.5 months (range 0–36 months). The median follow-up period was similar for both cohorts (BM: median 19.1 months, range 6.1–120.4 months; non-BM: median 27.2 months, range 6.2–53.4 months). Patients with BM vs non-BM had a similar distribution of disease subtype, visceral vs non-visceral disease, de-novo vs recurrent MBC, extracranial sites of disease, distribution of ethnicity (majority white women), and age at MBC diagnosis. Notably, there was a similar disease subtype distribution in BM (HR + /HER2:67%, HER2 + :11%, TNBC:17%) and non-BM (HR + /HER2-:69%, HER2 + :12%, TNBC:19%). The majority of patients with and without brain metastases were previously untreated for MBC at the time of cfDNA collection. Patients with brain metastases were treated with surgery alone (22%), surgery and radiation (5.5%), radiation alone (16%), radiation and systemic therapy (22%), systemic therapy alone (22%), and palliative care (11%). Median survival after the development of brain metastases was 11.6 months. Median overall survival after MBC diagnosis for BM was 26.4 months compared to 54.6 months for non-BM.

**Table 1:** Characteristics of patients with brain metastases (BM) versus those without brain metastases (non-BM).

Characteristic	BM (*n* = 18)	Non-BM (*n* = 68)	*p* value
Median time to development of BM after cfDNA testing (months)	11.5	N/A	N/A
Subtype			0.99
HR+/HER2−	12 (67%)	47 (69%)	
HER2+	2 (11%)	8 (12%)	
TNBC	3 (17%)	13 (19%)	
Unknown	1 (5.5%)		
Median age at MBC diagnosis (years)	58 Range: 42–64	61 Range: 52–68	0.18
Visceral Disease	12 (67%)	39 (57%)	0.47
Non-visceral disease	6 (33%)	29 (43%)	
Extracranial Sites of Disease at MBC diagnosis^a^			0.94
Liver	5 (28%)	22 (32%)	
Lung/pleura	8 (44%)	29 (43%)	
Bone	8 (44%)	38 (56%)	
Other	8 (44%)	40 (59%)	
De novo MBC	1 (5.5%)	8 (12%)	0.44
Recurrent MBC	17 (94%)	60 (88%)	
Ethnicity			0.75
White/Caucasian	15 (83%)	58 (85%)	
Asian	0 (0%)	3 (4%)	
Black/African American	1 (5.5%)	1 (1%)	
Other	1 (5.5%)	3 (4%)	
Unknown	1 (5.5%)	3 (4%)	
Patients with ≥1 detectable cfDNA mutation	17 (94%)	62 (91%)	0.65
MAF of most frequent mutation	Median 4.1%	Median 3.2%	0.57
	Mean 10.2%	Mean 9.7%	
Median number of mutations	4.5 (Range 0–14)	3 (0–12)	0.31

^a^Sites of disease are not mutually exclusive (thus columns do not add up to 100%).

### Genomic characteristics of BM vs non-BM

Of BM patients, 94% (17) had ≥1 detectable cfDNA mutation, while 91% (62) of non-BM patients had ≥1 detectable cfDNA mutation (*p* = 0.65). Patients with BM had a median of 4.5 mutations in baseline cfDNA compared to patients with non-BM who had a median of 3 (*p* = 0.31). Median number of mutations also did not vary significantly when corrected for disease subtype, possibly due to the small sample size (hormone receptor positive (HR+): BM median 2 mutations, non-BM 2; HER2+:BM 3, non-BM 2; triple negative breast cancer (TNBC): BM 3, non-BM 1). Patients with BM also had a median maximum mutant allele fraction (MAF) of 4.1% compared to 3.2% in the non-BM cohort.

**Fig. 2:**
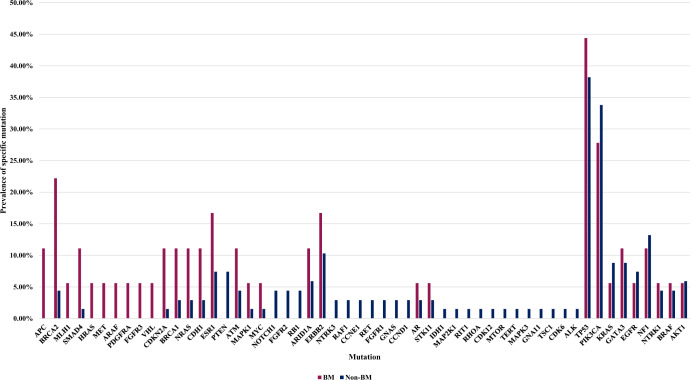
Mutation spectrum (baseline cfDNA) in patients with MBC who developed brain metastases during disease course (BM) vs those without brain metastases (non-BM). The mutation spectrum varied between the 2 cohorts as shown.

The mutation pattern varied between cohorts as depicted in Fig. 2. Notably, among patients who developed BM compared to non-BM, higher prevalence of *BRCA2* mutations (cases: 4/18 vs 3/68; percentage: 22.2% vs 4.4%, *p* = 0.01), *APC* mutations (cases: 2/18 vs 0/68; percentage: 11.1% vs 0.0%, *p* = 0.005), *SMAD4* mutations (cases: 2/18 vs 1/68; percentage: 11.1% vs 1.5%, *p* = 0.05) and *CDKN2A* mutations (cases 2/18 vs 1/68; percentage: 11.1% vs 1.5%, *p* = 0.05) were observed. Supplementary Table 1 compares the prevalence of individual mutations between BM and non-BM. A higher prevalence of *BRCA1* was seen in BM vs non-BM (11.1% vs 2.9%), although this difference is statistically nonsignificant (*p* = 0.14). Of the *BRCA1/2* mutations in this cohort, only 1 patient had a known germline *BRCA2* mutation (in BM cohort). Supplementary Table 2 classifies the mutations identified in the BM cohort as pathogenic, uncertain significance, or synonymous variants. Supplementary Fig. 1 compares frequencies of amplified genes between BM and non-BM; no statistically significant differences were seen between these cohorts.

Supplementary Fig. 2 depicts the mutation spectrum observed in 5 patients with brain metastases at the time of cfDNA testing at MBC diagnosis; notably, *BRCA1* (20%), *BRCA2* (20%), *CDKN2A* (40%), and *APC* (40%) were seen in cfDNA in this cohort.

**Fig. 3:**
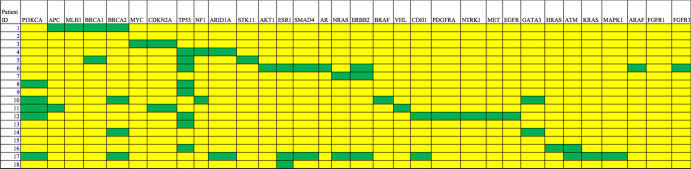
Cell-free DNA mutation spectrum in individual patients with BM. A statistically significant higher prevalence of *APC*, *BRCA2*, *CDKN2A*, and *SMAD4* mutations were seen in BM vs non-BM.

**Fig. 4:**
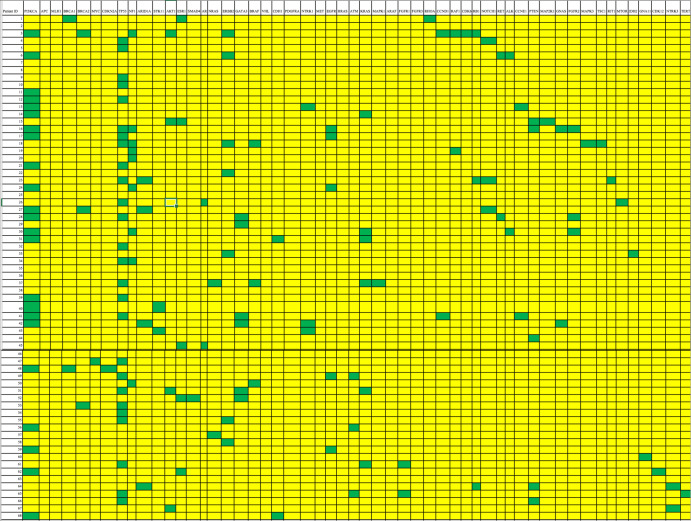
Cell-free DNA mutation spectrum in individual patients with non-BM. Differences in the mutation spectrum were seen between BM and non-BM.

The mutation spectrum for individual patients with BM or non-BM was then plotted, as depicted in Figs. 3, 4, to evaluate patterns of expression among patients with BM (Fig. 3) vs non-BM (Fig. 4). We were particularly interested in the expression of *APC*, *BRCA2*, *CKDN2A*, and *SMAD4* given the statistically significant higher prevalence seen in BM within the entire cohort. We observed that 7/18 patients who developed BM had at least 1 of the following 4 mutations present in baseline cfDNA: *APC, BRCA2, CDKN2A*, or *SMAD4* compared to only 5/68 patients with non-BM who had at least 1 of those 4 mutations (*p* = 0.001). Thus, this genomic pattern (having at least 1 of the following 4 mutations: *APC*, *BRCA2*, *CDKN2A*, or *SMAD4*) has a positive predictive value of 58%, negative predictive value of 85%, sensitivity of 39%, and specificity of 93% for prediction of early onset brain metastases in patients with MBC.

For patients with brain metastases, 5/18 (28%) underwent surgery or a brain biopsy. Due to the retrospective nature of this analysis, while we did not have brain tumor samples from all patients, four patients had tumor genotyping results available from the brain tumor specimens, with 3 of the cases demonstrating a concordant finding in cfDNA (at MBC diagnosis) and brain tumor tissue, as indicated in Supplementary Fig. 3. In the last case, the majority of mutations found on brain tumor tissue genotyping were not covered by the cfDNA panel sent at the time.

## Discussion

Brain metastases are associated with significant morbidity and mortality from MBC. A better understanding of risk factors for the development of brain metastases may enable the earlier identification and/or treatment of at-risk patients. While some features of breast tumors that predispose to the development of brain metastases are known, such as disease subtype, genomic predictors of brain metastases in patients with MBC are not well-delineated, given the inherent challenge of obtaining brain tumor tissue for genotyping. CfDNA can identify oncogenic mutations from DNA shed from tumor cells^14–18^. In this study, we compared cfDNA results collected at the time of MBC diagnosis in patients who developed brain metastases at or following MBC diagnosis with those who did not develop brain metastases.

Our results suggest that patients with MBC who subsequently develop early brain metastases may have different baseline cfDNA genomics. We observed a significantly higher prevalence of *APC*, *BRCA2, CDKN2A* and *SMAD4* mutations in patients who developed brain metastases than those without brain metastases.

The presence of differences in the genomic landscape of patients with MBC who go on to develop brain metastases is intriguing. As our median follow up period in both cohorts was about 2 years, the genomic differences we have observed may help to identify patients with MBC who may be at risk for the early development of brain metastases. Our finding of a higher prevalence of brain metastases in patients with cfDNA *APC, BRCA2, CDKN2A* or *SMAD4* mutations is intriguing, and merits further investigation in a larger prospective analysis controlling for disease subtype and presence of extracranial visceral disease. Interestingly, mutation burden was similar for BM vs non-BM in cfDNA, as was the presence of extracranial visceral disease, age at time of MBC diagnosis, and disease subtype in our cohort. Prior literature has suggested that germline *BRCA1/2* mutations may be associated with the development of brain metastases^19–21^. A study noted that CNS metastases frequently occurred in both *BRCA1* and *BRCA2* germline carriers (53% *BRCA1* and 50% *BRCA2* carriers), but only the *BRCA2* mutation had a statistically significant association with brain metastasis on multivariable analysis^20^. A second cohort also identified a high rate of brain metastases in patients with germline *BRCA1/2* mutations^22^. Although only 1 patient in our BM cohort was a known germline *BRCA2* carrier, it is possible that somatic *BRCA2* mutations may exhibit similar clinical behavior as germline *BRCA2* mutations^23^. Another study has demonstrated that brain metastases often demonstrate loss of the *APC* gene^24^. In addition, *CDKN2A* was found to be a commonly mutated gene in brain metastases from lung adenocarcinoma^25^, and a higher prevalence of *CDKN2A/p16* has also been observed in the lymph nodes of patients with breast cancer who developed brain metastases^26^. In lung adenocarcinoma, *SMAD4* has been detected in cerebrospinal fluid circulating tumor DNA^27^. *SMAD4* has also been implicated in the pathogenesis of breast cancer^28^.

Further prospective research is needed to validate the genomic pattern we have identified (i.e. presence of at least one of the 4 genes: *APC, BRCA2, CDKN2A* and *SMAD4*) that was more commonly observed in patients who developed brain metastases versus patients who did not experience brain metastases (7/18 vs 5/68, *p* = 0.001). Given the high specificity (93%) and negative predictive value (85%) of this genomic pattern in our cohort, this genomic pattern in cfDNA has the potential to serve as a genomic marker to identify patients with MBC who may have a lower risk of developing early brain metastases. As noted above, literature from other authors also lends support to the inclusion of *APC*, *BRCA2, CDKN2A*, and *SMAD4*. Indeed, this genomic pattern was identified in 3/5 patients with brain metastases at the time of MBC diagnosis in our cohort.

Future development of a preclinical model such as a cell line from a patient with MBC mutated with the genomic signature we identified could be used as a proof-of-concept of our hypothesis. Given the retrospective nature of our work, we were not able to conduct such an experiment in this study.

While the evaluation of cfDNA in patients with brain metastases is limited, prior studies have analyzed median levels of cfDNA in patients with glioblastoma or stage IV adenocarcinoma, demonstrating increases in cfDNA prior to tumor recurrence^29^. Other authors have also identified the presence of cfDNA mutations in patients with primary brain tumors^30^. Another approach that has been taken is to evaluate genomics using the CSF of patients with brain metastases^31–35^. The correlation of cfDNA and CSF findings is not well-established, and a potential direction for future research, since cfDNA offers the advantage of being a less invasive procedure. Research is also needed to help understand the correlation between cfDNA and brain tumor tissue mutation profiling; given our limited brain tissue sampling data, we were not able to study this correlation in our cohort, although concordance of genomic findings between baseline cfDNA at MBC diagnosis and brain tumor tissue genotyping was noted for 3 patients.

Additional limitations of our work include the retrospective nature of these analyses and modest sample size at a single institution limiting further subset analyses. Furthermore, as we used the Guardant360® platform, we are limited to the genotyping panel included in this assay. Using whole exome sequencing could potentially identify additional mutations of potential significance.

Our finding that the mutation spectrum of cfDNA collected at the time of MBC diagnosis may vary in patients who subsequently develop early brain metastases warrants further investigation. The prospective validation of genomic predictors using cfDNA could potentially guide precision medicine interventions to decrease the risk of developing brain metastases and/or support enhanced screening measures to enable earlier identification of brain metastases in high-risk populations.

## Methods

This study was performed in compliance with the Declaration of Helsinki. The retrospective analyses were performed with IRB approval from an institutional protocol. Per IRB regulations, individual patient consent was not required for this retrospective analysis, although all patients had been consented for Guardant360® cfDNA testing prior to collection.

### cfDNA

The cfDNA analysis was conducted as part of routine clinical practice via the Guardant360® assay (Guardant Health Inc., Palo Alto, CA). cfDNA was obtained from whole blood, with blood draw, shipment, plasma isolation, and cfDNA extraction procedures previously described^36^. Guardant360® is CLIA-certified, College of American Pathologists-accredited, New York State Department of Health-approved cfDNA next-generation sequencing (NGS) assay with analytical and clinical validation previously reported^17,36,37^. At the time of the study, Guardant360® included analysis of single nucleotide variants (SNVs) in 73- to 74-genes (the assay evolved over the course of the study period), as well as small insertions/deletion (indels), copy number amplifications, and gene rearrangement/fusions in a subset of genes. The reportable range for SNVs, indels, fusions, and CNAs is ≥0.04%, ≥0.02%, ≥0.04%, and ≥2.12 copies, respectively, with >99.9999% per-position analytic specificity^36^.

### Comparison of baseline cfDNA genomics (at time of MBC diagnosis) in patients with MBC who developed brain metastases (BM) vs those without brain metastases (non-BM)

#### Patient population

In this study, we identified patients with MBC at the Massachusetts General Hospital who underwent cfDNA testing (detailed above) as part of routine clinical care at the time of MBC diagnosis between 1/2016–10/2019 with greater than or equal to 6 months of follow-up at our institution post-testing. From this cohort of patients, the subset of patients who developed brain metastases either at the time of or after cfDNA testing was identified. A retrospective review of medical records and pathology reports was conducted to determine demographics and tumor subtype. In addition, a retrospective review of Guardant360® reports was conducted to analyze the cfDNA for the number and type of mutations, as well as the maximum mutant allele fraction (MAF). For patients for whom brain tumor tissue was available, a comparison of the cfDNA results at MBC diagnosis with the brain tumor genotyping results was made.

#### Analysis

Clinical and genomic features of patients who developed brain metastases (BM) at or after cfDNA collection at MBC diagnosis, and those without brain metastases (non-BM) were compared using Pearson’s and Wilcoxon rank sum tests, with *p* ≤ 0.05 for statistical significance. Fisher’s exact test with *p* ≤ 0.05 for statistical significance was used to compare gene amplifications between BM vs non-BM.

A comparison of the mutational spectrum in patients who developed brain metastases versus those who did not was performed to help identify a panel of genes that might risk stratify patients for the development of brain metastases.

### Reporting summary

Further information on research design is available in the Nature Research Reporting Summary linked to this article.

## Supplementary information

Supplementary Material

Reporting Summary

## Data Availability

Data used in this study is not publically available as it contains patient information including genomic/genetic information, which cannot be released as this is not covered by the informed consent signed by patients. Clarifications on the study data may be requested in writing to the corresponding author.
