# Supplementary material for: Utilizing cell-free DNA to predict risk of developing brain metastases in patients with metastatic breast cancer
Source: NPJ Breast Cancer. 2023 Apr 19;9:29. doi: 10.1038/s41523-023-00528-z (PMC10115848; doi:10.1038/s41523-023-00528-z)
Supplement: Supplementary file 1 — Supplementary Material [file 41523_2023_528_MOESM1_ESM.pdf]

Supplementary Figure 1. Comparison of gene amplifications in cell-free DNA between BM vs. non-BM.

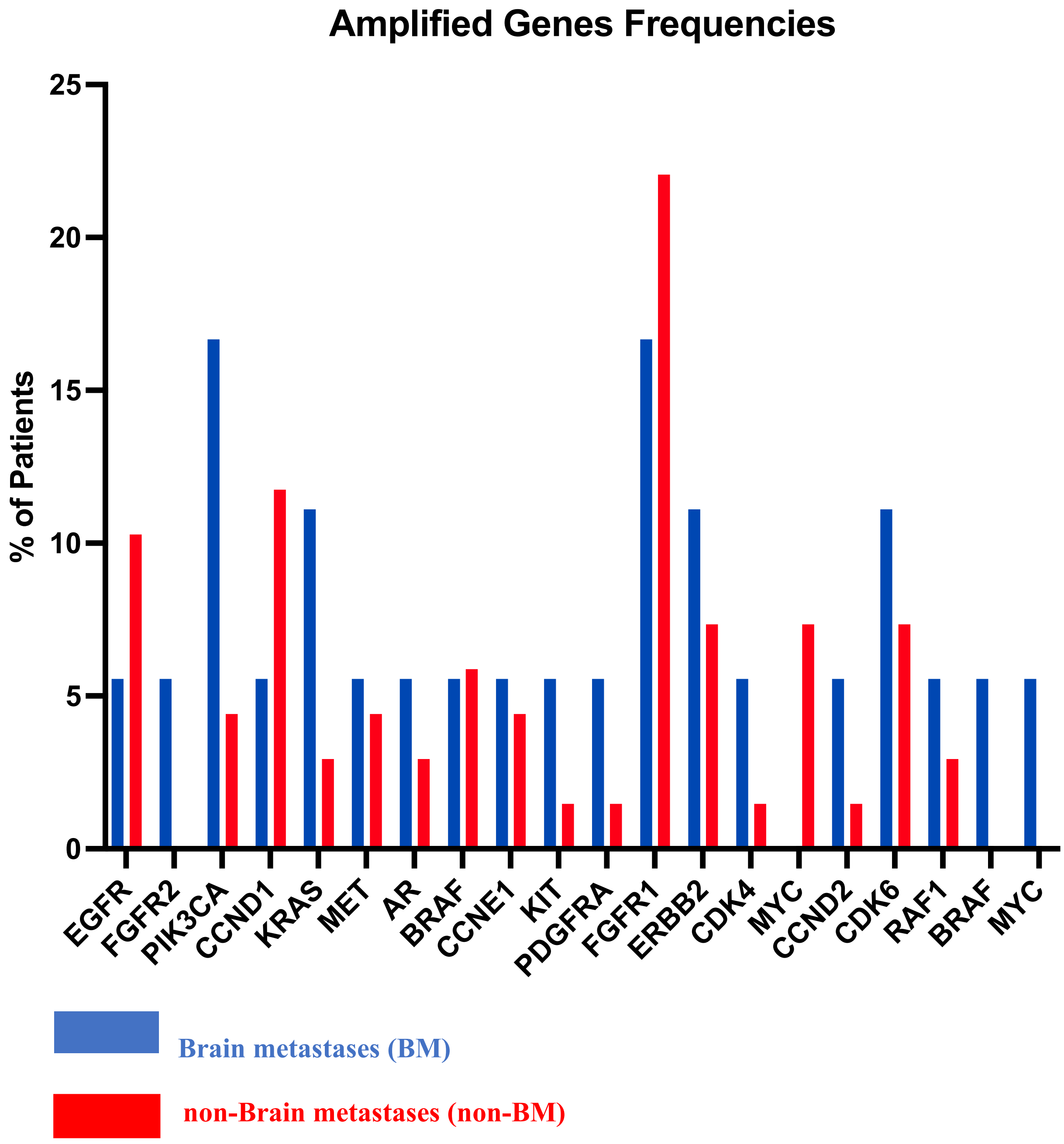

Supplementary Figure 2. cfDNA Mutation spectrum in 5 patients with brain metastases at MBC diagnosis.

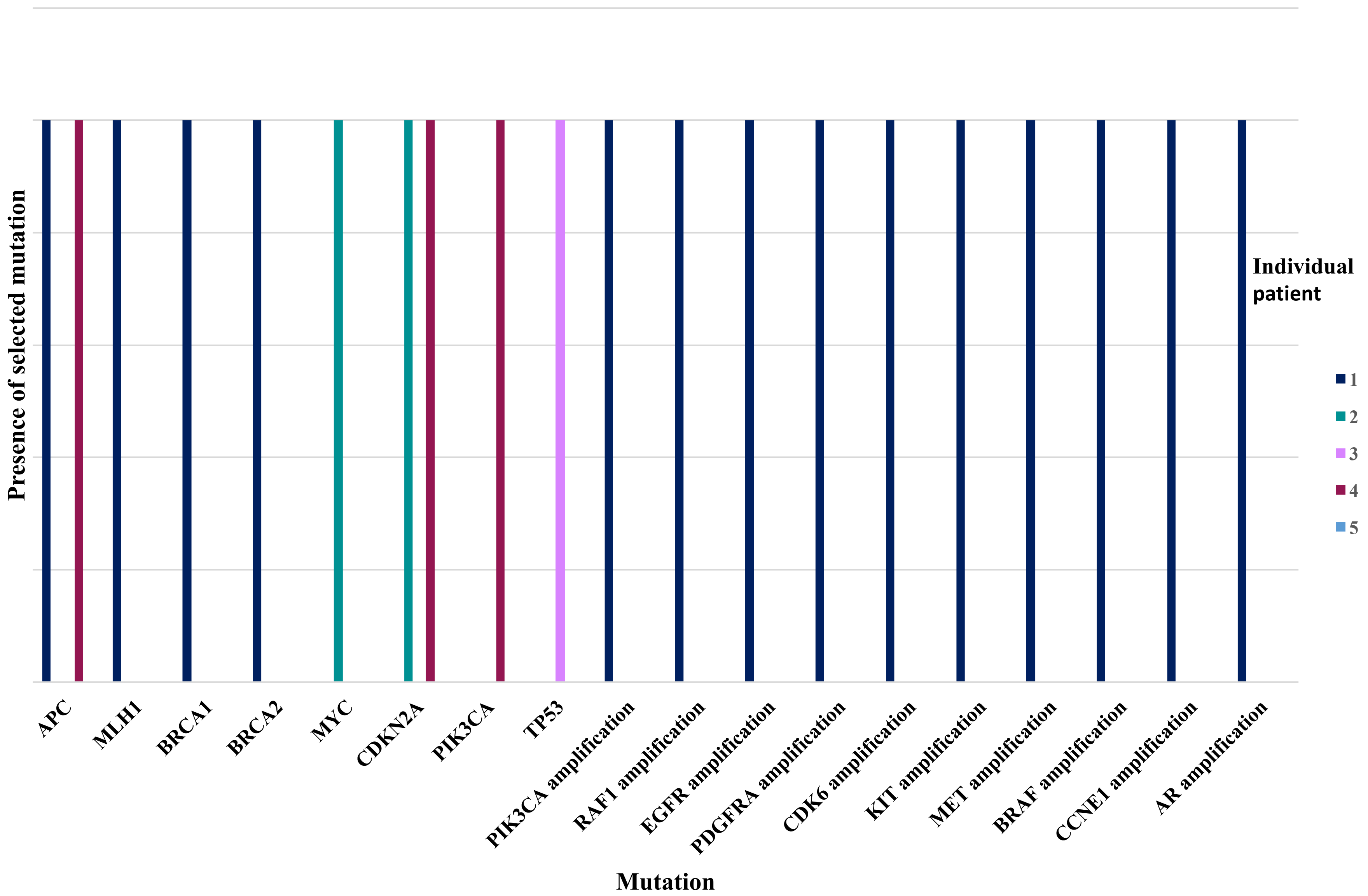

Vidula N et al.

Supplementary Figure 3. Breast cancer history and mutation spectrum in patients who underwent resection of brain metastases.

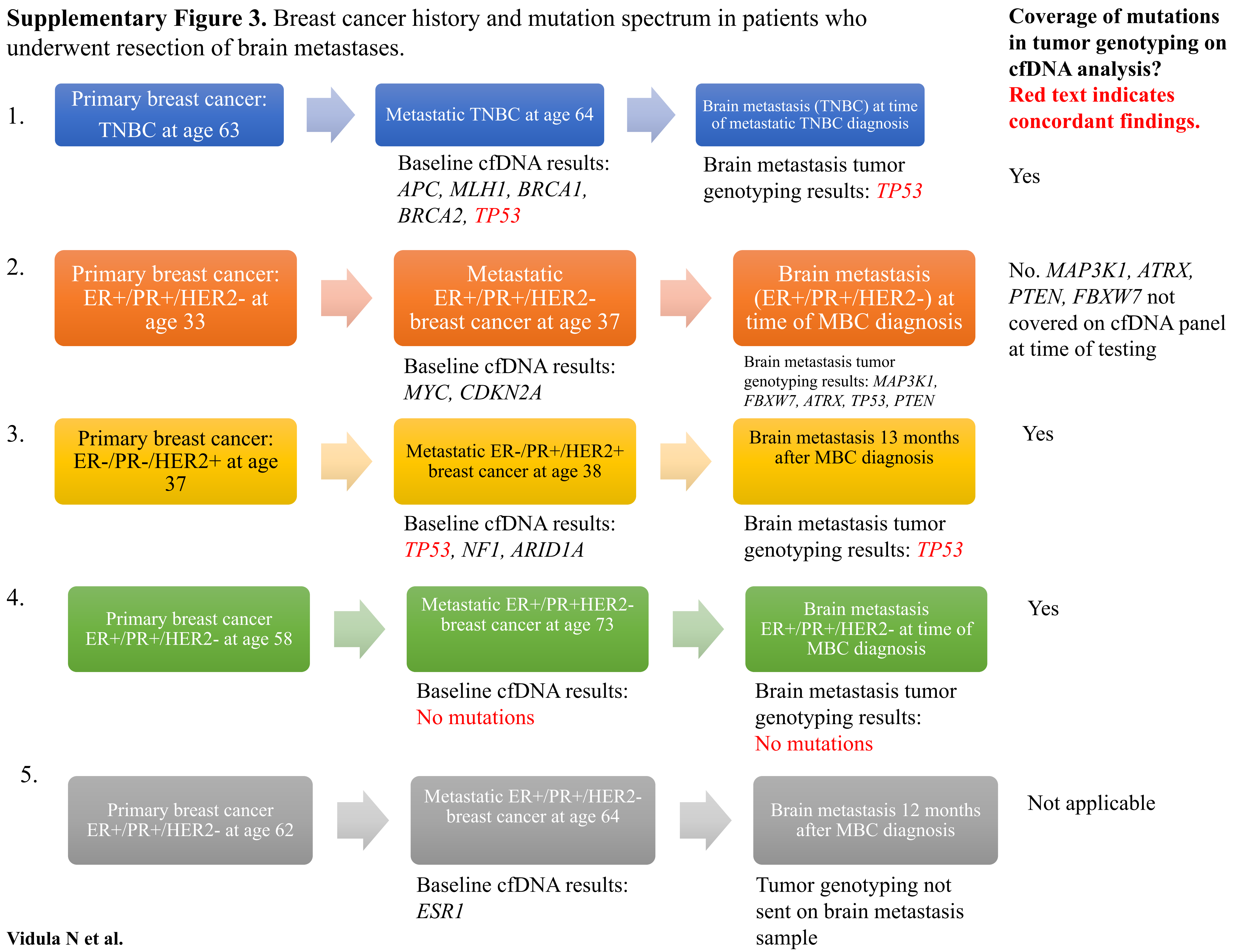

Vidula N et al.

Supplementary Table 1. Mutation spectrum in BM vs non-BM.

Mutation	BM	Non-BM	p-value
APC	2 (11.1%)	0 (0.0%)	.005
BRCA2	4 (22.2%)	3 (4.4%)	.01
MLH1	1 (5.6%)	0 (0.0%)	.05
SMAD4	2 (11.1%)	1 (1.5%)	.05
HRAS	1 (5.6%)	0 (0.0%)	.05
MET	1 (5.6%)	0 (0.0%)	.05
ARAF	1 (5.6%)	0 (0.0%)	.05
PDGFRA	1 (5.6%)	0 (0.0%)	.05
FGFR3	1 (5.6%)	0 (0.0%)	.05
VHL	1 (5.6%)	0 (0.0%)	.05
CDKN2A	2 (11.1%)	1 (1.5%)	.05
BRCA1	2 (11.1%)	2 (2.9%)	.14
NRAS	2 (11.1%)	2 (2.9%)	.14
CDH1	2 (11.1%)	2 (2.9%)	.14
ESR1	3 (16.7%)	5 (7.4%)	.23
PTEN	0 (0.0%)	5 (7.4%)	.24
ATM	2 (11.1%)	3 (4.4%)	.28
MAPK1	1 (5.6%)	1 (1.5%)	.31
MYC	1 (5.6%)	1 (1.5%)	.31
NOTCH1	0 (0.0%)	3 (4.4%)	.36
FGFR2	0 (0.0%)	3 (4.4%)	.36
RB1	0 (0.0%)	3 (4.4%)	.36
ARID1A	2 (11.1%)	4 (5.9%)	.44
ERBB2	3 (16.7%)	7 (10.3%)	.45
NTRK3	0 (0.0%)	2 (2.9%)	.46
RAF1	0 (0.0%)	2 (2.9%)	.46
CCNE1	0 (0.0%)	2 (2.9%)	.46
RET	0 (0.0%)	2 (2.9%)	.46
FGFR1	0 (0.0%)	2 (2.9%)	.46
GNAS	0 (0.0%)	2 (2.9%)	.46
CCND1	0 (0.0%)	2 (2.9%)	.46
AR	1 (5.6%)	2 (2.9%)	.59
STK11	1 (5.6%)	2 (2.9%)	.59
IDH1	0 (0.0%)	1 (1.5%)	.6
MAP2K1	0 (0.0%)	1 (1.5%)	.6
RIT1	0 (0.0%)	1 (1.5%)	.6
RHOA	0 (0.0%)	1 (1.5%)	.6
CDK12	0 (0.0%)	1 (1.5%)	.6
MTOR	0 (0.0%)	1 (1.5%)	.6
TERT	0 (0.0%)	1 (1.5%)	.6
MAPK3	0 (0.0%)	1 (1.5%)	.6
GNA11	0 (0.0%)	1 (1.5%)	.6
TSC1	0 (0.0%)	1 (1.5%)	.6
CDK6	0 (0.0%)	1 (1.5%)	.6
ALK	0 (0.0%)	1 (1.5%)	.6
TP53	8 (44.4%)	26 (38.2%)	.63
PIK3CA	5 (27.8%)	23 (33.8%)	.63
KRAS	1 (5.6%)	6 (8.8%)	.65
GATA3	2 (11.1%)	6 (8.8%)	.77
EGFR	1 (5.6%)	5 (7.4%)	.79
NF1	2 (11.1%)	9 (13.2%)	.81
NTRK1	1 (5.6%)	3 (4.4%)	.84
BRAF	1 (5.6%)	3 (4.4%)	.84
AKT1	1 (5.6%)	4 (5.9%)	.96

Supplementary Table 2. Classification of mutations in cfDNA of patients with BM as pathogenic, uncertain significance, or synonymous variants.

Patient ID	cfDNA Results
1	**APC S2114T**; *MLH1 A442A*; *BRCA1 P364P*; **BRCA2 E2364Q**; PIK3CA Amplification; RAF1 Amplification; EGFR Amplification; PDGFRA Amplification; CDK6 Amplification; KIT Amplification; MET Amplification; BRAF Amplification; CCNE1 Amplification; AR Amplification
2	PIK3CA Amplification
3	**MYC D173A**; **CDKN2A L130R**
4	TP53 P82fs; TP53 A88fs; NF1 K1585fs; **ARID1A G2087R**; ERBB2 Amplification
5	TP53 R248Q, TP53 Exon 4 Deletion, BRCA1 Splice Site SNV, STK11 Y292*, TP53 R273H, TP53 Splice Site SNV, TP53 R213L, TP53 I195T, TP53 Q317*, TP53 H179Y, TP53 V272M, TP53 H179R, TP53 V143M
6	AKT1 E17K, ESR1 D538G, SMAD4 Q442*, SMAD4 Q180*, SMAD4 Q183*, *AR G373G*, **FGFR3 K404N**, *ERBB2 R1153R*, **ARAF T213P**, SMAD4 S242, TP53 R248Q
7	NRAS G12D, **ERBB2 P36L**, ERBB2 Amplification
8	PIK3CA E545K; TP53 V272M; CDK4 Amplification; KRAS Amplification; FGFR1 Amplification
9	TP53 R249W
10	PIK3CA E545K; GATA3 R331fs; *BRCA2 P606P*; *BRAF T241T*; **NF1 R135W**
11	PIK3CA E545K; **CDKN2A S152L**; APC N1792fs; VHL E94*
12	PIK3CA Q546R; **PIK3CA E737Q**; CDH1 S70fs; TP53 R280T; PDGFRA Splice Site SNV; **PIK3CA E78K**; *NTRK1 I572I*; *MET L262L*; **EGFR E400K**; KRAS Amplification; CCND1 Amplification; CDK6 Amplification; BRAF Amplification
13	TP53 Splice Site SNV; FGFR2 Amplification
14	BRCA2 Splice Site SNV; FGFR1 Amplification; GATA3 P409fs
15	No mutations/alterations
16	TP53 H179R; *HRAS K104K*; **ATM L1956R**; CCND2 Amplification; MYC Amplification
17	ARID1A S1791*; PIK3CA E545K; PIK3CA E726K; **SMAD4 G352A**; CDH1 E353fs; **NRAS S145L**; **ATM E2932D**; **ESR1 Q314K**; **ERBB2 R599C**; KRAS A146V; MAPK1 S142L; **BRCA2 V572L**
18	ESR1 D538G; ESR1 Y537C; FGFR1 Amplification; PIK3CA Amplification

Bold=variants of uncertain significance, *italic=synonymous variants,* and plain text=pathogenic
