# Supplementary material for: Utilizing cell-free DNA to predict risk of developing brain metastases in patients with metastatic breast cancer
Source: NPJ Breast Cancer. 2023 Apr 19;9:29. doi: 10.1038/s41523-023-00528-z (PMC10115848; doi:10.1038/s41523-023-00528-z)
Supplement: Supplementary file 2 — Reporting Summary [file 41523_2023_528_MOESM2_ESM.pdf]

nature portfolio

Corresponding author(s): Neelima Vidula

Last updated by author(s): 6/21/2022

Reporting Summary

Nature Portfolio wishes to improve the reproducibility of the work that we publish. This form provides structure for consistency and transparency in reporting. For further information on Nature Portfolio policies, see our Editorial Policies and the Editorial Policy Checklist.

Statistics

For all statistical analyses, confirm that the following items are present in the figure legend, table legend, main text, or Methods section.

n/a	Confirmed	
☐	☒	The exact sample size (*n*) for each experimental group/condition, given as a discrete number and unit of measurement
☒	☐	A statement on whether measurements were taken from distinct samples or whether the same sample was measured repeatedly
☐	☒	The statistical test(s) used AND whether they are one- or two-sided<br>*Only common tests should be described solely by name; describe more complex techniques in the Methods section.*
☒	☐	A description of all covariates tested
☒	☐	A description of any assumptions or corrections, such as tests of normality and adjustment for multiple comparisons
☒	☐	A full description of the statistical parameters including central tendency (e.g. means) or other basic estimates (e.g. regression coefficient) AND variation (e.g. standard deviation) or associated estimates of uncertainty (e.g. confidence intervals)
☒	☐	For null hypothesis testing, the test statistic (e.g. *F*, *t*, *r*) with confidence intervals, effect sizes, degrees of freedom and *P* value noted<br>*Give P values as exact values whenever suitable.*
☒	☐	For Bayesian analysis, information on the choice of priors and Markov chain Monte Carlo settings
☒	☐	For hierarchical and complex designs, identification of the appropriate level for tests and full reporting of outcomes
☒	☐	Estimates of effect sizes (e.g. Cohen's *d*, Pearson's *r*), indicating how they were calculated

Our web collection on statistics for biologists contains articles on many of the points above.

Software and code

Policy information about availability of computer code

Data collection	N/A
Data analysis	N/A

For manuscripts utilizing custom algorithms or software that are central to the research but not yet described in published literature, software must be made available to editors and reviewers. We strongly encourage code deposition in a community repository (e.g. GitHub). See the Nature Portfolio guidelines for submitting code & software for further information.

Data

Policy information about availability of data

All manuscripts must include a data availability statement. This statement should provide the following information, where applicable:

- Accession codes, unique identifiers, or web links for publicly available datasets
- A description of any restrictions on data availability
- For clinical datasets or third party data, please ensure that the statement adheres to our policy

Data used in this study is not publically available as it contains patient information including genomic information. However, de-identified data can be provided upon reasonable request.

Human research participants

Policy information about studies involving human research participants and Sex and Gender in Research.

Reporting on sex and gender	This study was a retrospective study of patients with breast cancer. Patients were not excluded based on gender.
Population characteristics	In this study, we identified patients at the Massachusetts General Hospital who underwent cfDNA testing (Guardant360®, next generation sequencing, 73 gene assay) as part of routine clinical care at the time of MBC diagnosis between 1/2016-10/2019 with greater than or equal to 6 months of follow-up at our institution post-testing. From this cohort of patients, the subset of patients who developed brain metastases either at the time of or after cfDNA testing were identified.
Recruitment	Patients were not recruited as this was a retrospective analysis.
Ethics oversight	This study was performed in compliance with the Declaration of Helsinki. The retrospective analyses were performed with IRB approval from an institutional protocol. Per IRB regulations, individual patient consent was not required for this retrospective analysis, although all patients had been consented for Guardant360� cfDNA testing prior to collection.

Note that full information on the approval of the study protocol must also be provided in the manuscript.

Field-specific reporting

Please select the one below that is the best fit for your research. If you are not sure, read the appropriate sections before making your selection.

☒ Life sciences ☐ Behavioural & social sciences ☐ Ecological, evolutionary & environmental sciences

For a reference copy of the document with all sections, see nature.com/documents/nr-reporting-summary-flat.pdf

Life sciences study design

All studies must disclose on these points even when the disclosure is negative.

Sample size	In this study, we identified patients at the Massachusetts General Hospital who underwent cfDNA testing (Guardant360®, next generation sequencing, 73 gene assay) as part of routine clinical care at the time of MBC diagnosis between 1/2016-10/2019 with greater than or equal to 6 months of follow-up at our institution post-testing. From this cohort of patients, the subset of patients who developed brain metastases either at the time of or after cfDNA testing were identified.
Data exclusions	No patients meeting the above criteria were excluded.
Replication	N/A
Randomization	N/A
Blinding	N/A

Reporting for specific materials, systems and methods

We require information from authors about some types of materials, experimental systems and methods used in many studies. Here, indicate whether each material, system or method listed is relevant to your study. If you are not sure if a list item applies to your research, read the appropriate section before selecting a response.

Materials & experimental systems

n/a	Involved in the study	
☒	☐	Antibodies
☒	☐	Eukaryotic cell lines
☒	☐	Palaeontology and archaeology
☒	☐	Animals and other organisms
☐	☒	Clinical data
☒	☐	Dual use research of concern

Methods

n/a	Involved in the study	
☒	☐	ChIP-seq
☒	☐	Flow cytometry
☒	☐	MRI-based neuroimaging

Clinical data

Policy information about clinical studies

All manuscripts should comply with the ICMJE guidelines for publication of clinical research and a completed CONSORT checklist must be included with all submissions.

Clinical trial registration	N/A-This is not a clinical trial.
Study protocol	N/A- retrospective research (see above)
Data collection	N/A- Retrospective data collection from institutional cohort.
Outcomes	N/A- Retrospective analysis of various demographic variables.
